# Endoscopic prediction model for differentiating upper submucosal invasion (< 200 μm) and beyond in superficial esophageal squamous cell carcinoma

**DOI:** 10.18632/oncotarget.23900

**Published:** 2018-01-03

**Authors:** Joohwan Bae, In Seub Shin, Yang Won Min, Insuk Sohn, Joong Hyun Ahn, Hyuk Lee, Byung-Hoon Min, Jun Haeng Lee, Poong-Lyul Rhee, Jae J. Kim

**Affiliations:** ^1^ Department of Medicine, Samsung Medical Center, Sungkyunkwan University School of Medicine, Seoul, Korea; ^2^ Biostatistics and Clinical Epidemiology Center, Research Institute for Future Medicine, Samsung Medical Center, Sungkyunkwan University School of Medicine, Seoul, Korea

**Keywords:** depth, endoscopic submucosal dissection, endoscopy, superficial esophageal squamous cell carcinoma, prediction

## Abstract

Esophageal endoscopic submucosal dissection (ESD) can be attempted in superficial esophageal squamous cell carcinoma (SESCC) invading the upper submucosal layer (SM1: invasion < 200 μm). This study aimed to determine endoscopic predictive features associated with beyond SM1 invasion in SESCC and establish a predictive model using the identified features. This study retrospectively analyzed 203 esophageal ESD for SESCC. Endoscopic images were reviewed by two endoscopists. Tumors were evaluated for main shape, sizes, and surface characteristics. The association between each endoscopic feature and beyond SM1 invasion was evaluated. Using the significant endoscopic features in multivariate analysis, a predictive model for beyond SM1 invasion in SESCC was established. Among 203 SESCCs, 40 (19.7%) invaded beyond SM1. Multivariate analysis revealed that surface nodularity [odds ratio (OR) 41.340, 95% confidence interval (CI) 8.492–201.252, *p* < 0.001], surface granularity (OR 18.682, 95% CI 4.818–72.440, *p* < 0.001), surface unevenness, (OR 4.107, 95% CI 1.160–14.543, *p* = 0.029), deep depression (OR 27.490, 95% CI 2.897–260.853, *p* = 0.004), and thick notch (OR 41.701, 95% CI 6.646–261.672, *p* < 0.001) were independently associated with beyond SM1 invasion. An established model showed an area under the curve of 0.921 with 95% CI 0.881–0.960. The best cut-off value showed the following: sensitivity, 0.85; specificity, 0.83; positive predictive value, 0.55; and negative predictive value, 0.96. In conclusion, endoscopic features can predict beyond SM1 invasion in SESCC. Our prediction model is potentially useful for screening ESD candidates in SESCC.

## INTRODUCTION

Esophageal cancer cases have increased due to aging and the increasing population [1]. Superficial esophageal squamous cell carcinoma (SESCC) is frequently detected with screening endoscopy, and the development of endoscopic imaging techniques facilitates early diagnosis [2–6]. Endoscopic resection has been used for SESCC when a negligible risk of lymph node metastasis (LNM) exists [7, 8]. The risk of LNM increases according to the depth of SESCC [9–11]. Thus, guidelines state that cancers confined to the mucosa or lamina propria are indicated for esophageal endoscopic resection, preferably endoscopic submucosal dissection (ESD) [12–15]. However, clinical determination of depth of tumor invasion is often difficult [16, 17]. In addition, the presence of lymphovascular invasion (LVI), which is a strong risk factor for LNM, cannot be investigated prior to endoscopic resection.

Given the low sensitivity of current imaging modalities for LNM [18], ESD for SESCC has a role not only for local treatment but also for exact LNM risk stratification. As esophagectomy carries a considerable morbidity and mortality [19–21], SESCC invading less than submucosal invasion < 200 μm (SM1) could be treated using ESD with favorable long-term outcomes [13, 22–24]. Thus, ESD should be tried unless there is obvious evidence of deep SM invasion. However, if we could differentiate within SM1 invasion from SM2 invasion, unnecessary procedure either esophagectomy or ESD would be minimized.

Endoscopic appearance is associated with depth of SESCC invasion [25]. A recent study has shown a comparable accuracy for predicting depth of invasion between conventional endoscopy and magnifying endoscopy (ME) with narrow-band imaging (NBI) [17]. However, calculating the sum of objective risks for deep invasion from several endoscopic tumor features is difficult. If calculated, endoscopic prediction for depth of tumor invasion could be better and easier than before.

Thus, in this study, we aimed to investigate endoscopic predictive features associated with SESCC invading beyond SM1 and establish a mathematic prediction model for beyond SM1 invasion in SESCC.

## RESULTS

### Baseline characteristics of subjects and tumors

The baseline characteristics of the study subjects and endoscopic findings are summarized in Table 1. Among 203 SESCCs, 163 (80.3%) were within SM1 and 40 (19.7%) invaded beyond SM1. The beyond SM1 group had a higher age than the within SM1 group (67.6 ± 7.3 years vs. 64.2 ± 8.0 years, *p* = 0.016), but no differences were found regarding sex and body mass index. IIb (a flat lesion) or IIc (slightly depressed lesion) main shape was more frequently observed in the within SM1 group, and Is (a sessile lesion) or IIa (a slightly elevated lesion) main shape was more frequently observed in the beyond SM1 group. All three tumor sizes were larger in the beyond SM1 group than in the within SM1 group. Surface nodularity (gross protuberance size > 5 mm) and granularity (gross protuberance size between 2 and 5 mm), multiple elevated foci (more than 3 foci of surface nodularity or granularity), deep depression (deeper than IIc), and thick notch (V-shaped notch with surrounding thickened margin) were more frequently observed in the beyond SM1 group than in the within SM1 group. Surface unevenness (gross protuberance size < 2 mm) was similarly observed between the two groups. Well-differentiated histology was more frequently observed in the within SM1 group than in the beyond SM1 group, whereas poor differentiation was only observed in the beyond SM1 group. The most common depth of tumor invasion in the within SM1 group was M2 (limited to the lamina propria, 46.6%), followed by M3 (limited to the muscularis mucosa, 33.1%), M1 (limited to the intraepithelium, 15.3%), and SM1 (4.9%). LVI was more frequently observed in the beyond SM1 group than in the within SM1 group (40.0% vs. 5.5%, *p* < 0.001).

**Table 1:** Baseline characteristics and endoscopic findings of study subjects according to the depth of tumor invasion

Variable	Within SM1 (*n* = 163)	Beyond SM1 (*n* = 40)	*p* value
Age (years)	64.2 ± 8.0	67.6 ± 7.3	0.016
Male	151 (92.6)	38 (95.0)	0.857
BMI (kg/m^2^)	23.8 ± 3.4	24.3 ± 3.0	0.411
Main shape			< 0.001
Is	1 (0.6)	8 (20.0)	
IIa	11 (6.7)	8 (20.0)	
IIb	120 (73.6)	19 (47.5)	
IIc	31 (19.0)	5 (12.5)	
Long tumor size (mm)	17.5 ± 12.1	22.4 ± 14.7	0.029
Short tumor size (mm)	12.8 ± 8.0	16.8 ± 8.3	0.006
2-dimensional tumor size (mm^2^)	297.3 ± 417.8	460.2 ± 510.4	0.036
Surface nodularity	6 (3.7)	9 (22.5)	< 0.001
Surface granularity	18 (11.0)	13 (32.5)	0.002
Surface unevenness	40 (24.5)	12 (30.0)	0.612
Multiple elevated foci	9 (5.5)	10 (25.0)	< 0.001
Deep depression	2 (1.2)	7 (17.5)	< 0.001
Thick notch	2 (1.2)	12 (30.0)	< 0.001
Tumor differentiation			0.004
Well differentiation	48 (29.4)	6 (15.0)	
Moderate differentiation	115 (70.6)	32 (80.0)	
Poor differentiation	0 (0.0)	2 (5.0)	
Tumor depth			< 0.001
M1	25 (15.3)	0 (0.0)	
M2	76 (46.6)	0 (0.0)	
M3	54 (33.1)	0 (0.0)	
SM1	8 (4.9)	0 (0.0)	
Beyond SM1	0 (0.0)	40 (100.0)	
LVI	9 (5.5)	16 (40.0)	< 0.001

Data are shown as the mean ± SD or number (%) of patients. SM1, submucosal invasion < 200 μm; BMI, body mass index; M1, intraepithelium; M2, lamina propria; M3, muscularis mucosa; LVI, lymphovascular invasion.

### Endoscopic appearances associated with beyond SM1 invasion

Multivariate analysis revealed that surface nodularity [Odds ratio (OR) 41.340, 95% Confidence interval (CI) 8.492–201.252, *p* < 0.001], surface granularity (OR 18.682, 95% CI 4.818–72.440, *p* < 0.001), surface unevenness, (OR 4.107, 95% CI 1.160–14.543, *p* = 0.029), deep depression (OR 27.490, 95% CI 2.897–260.853, *p* = 0.004), and thick notch (OR 41.701, 95% CI 6.646–261.672, *p* < 0.001) were independently associated with beyond SM1 invasion (Table 2). Main shape was also associated with beyond SM1 invasion with a marginal statistical significance (*p* = 0.070).

**Table 2:** Multivariate selection analysis of the association between endoscopic characteristics and beyond SM1 invasion in superficial esophageal squamous cell carcinomas

Variable	Estimate	OR (95% CI)	*p* value
Main shape			0.070
Is	2.8778	17.776 (0.708–446.406)	
IIa	1.5506	4.714 (1.104–20.137)	
IIb	-	-	
IIc	−0.2174	0.805 (0.182–3.558)	
Surface nodularity	3.7218	41.340 (8.492–201.252)	< 0.001
Surface granularity	2.9276	18.682 (4.818–72.440)	< 0.001
Surface unevenness	1.4126	4.107 (1.160–14.543)	0.029
Deep depression	3.3138	27.490 (2.897–260.853)	0.004
Thick notch	3.7305	41.701 (6.646–261.672)	< 0.001

SM1, submucosal invasion < 200 μm; OR, odds ratio; CI, confidence interval.

### Prediction model for beyond SM1 invasion

**Figure 1:**
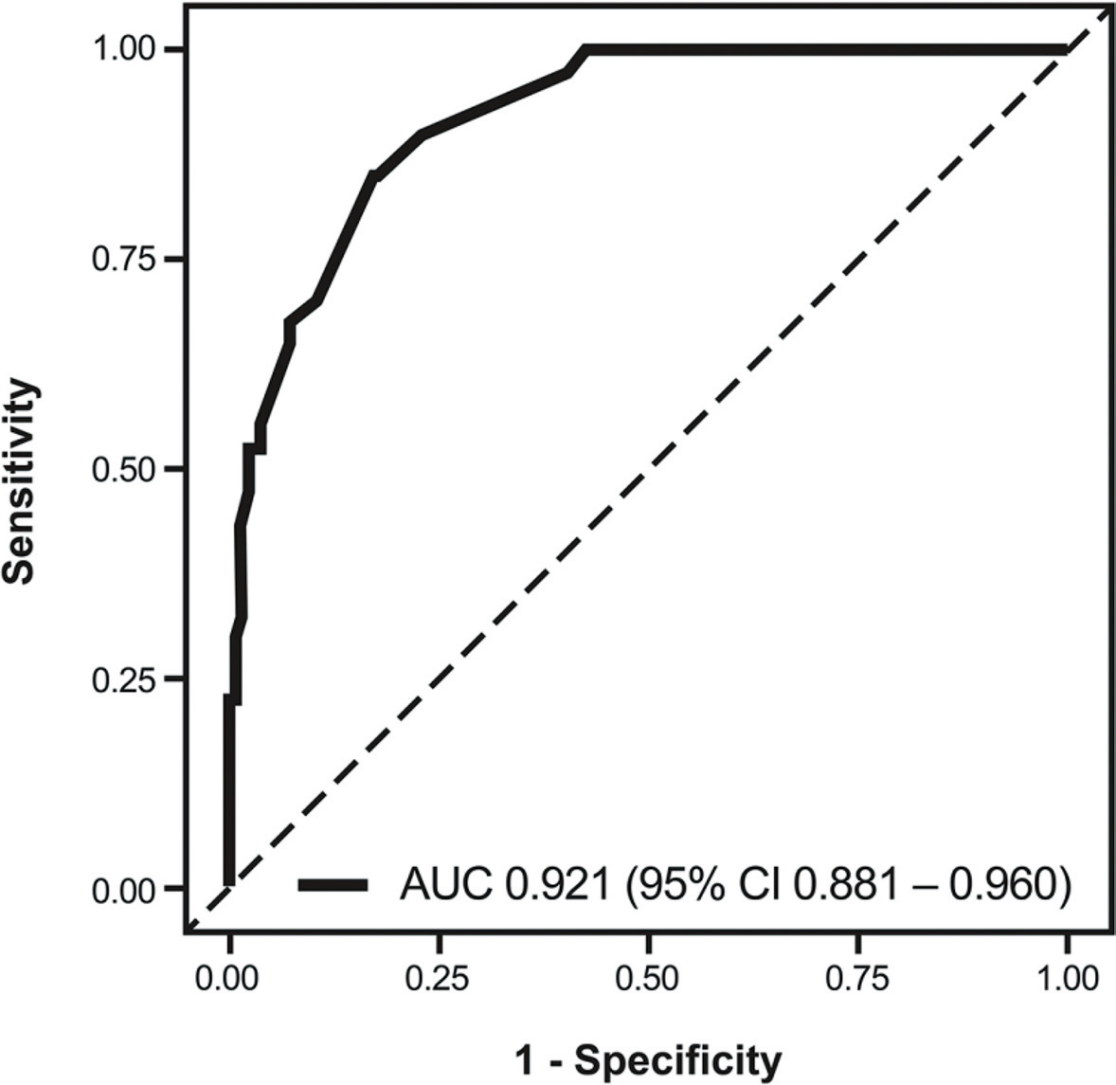
Receiver operating characteristic curve of the prediction model for beyond SM1 invasion in SESCC The area under receiver operating characteristics curve was 0.921 (95% CI, 0.881–0.960). SM1, submucosal invasion < 200 μm; SESCC, superficial esophageal squamous cell carcinoma; CI, confidence interval.

**Figure 2:**
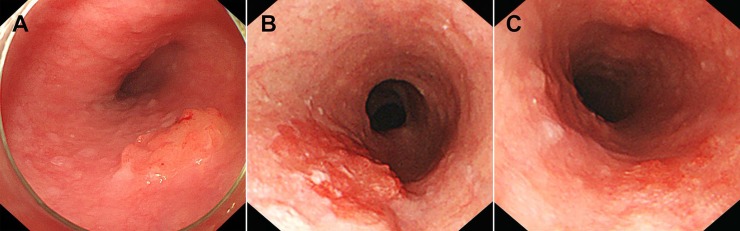
Representative examples of the prediction model (**A**) A 1.5 cm-sized flat elevated lesion with nodular protuberances of more than 5 mm and granular protuberances of between 2 mm and 5 mm on the surface. This lesion corresponds to IIa of 47 risk points, surface nodularity of 100 risk points, and surface granularity of 78 risk points. Since the total point (225) is more than 84, the depth of tumor is expected to be beyond SM1. The pathologic depth of tumor was 2000 μm below the muscularis mucosa. (**B**) A 2.5cm-sized flat lesion with granular protuberances of between 2 mm and 5 mm on the surface. This lesion corresponds to IIb of 6 risk points and surface granularity of 78 risk points. Since the total point is 84, the depth of tumor is expected to be between SM1 and SM2. The pathologic depth of tumor was 200 μm below the muscularis mucosa. (**C**) A 1 cm-sized flat lesion with uneven surface. This lesion corresponds to IIb of 6 risk points and surface unevenness of 38 risk points. Since the total point (44) is less than 84, the depth of tumor is expected to be within SM1. Pathologic depth of tumor was muscularis mucosa. SM1, submucosal invasion < 200 μm; SM2, beyond SM1 (submucosal invasion ≥ 200 μm).

Risk points of each endoscopic characteristic for beyond SM invasion were calculated. The risk points attributed to each risk factor were weighted according to respective coefficients in multivariate logistic regression (Table 2). The risk points were used to establish a mathematical prediction model for beyond SM1 invasion. The equation of prediction model is as follows:

Total risk points of beyond SM1 invasion = (83 × Is) + (47 × IIa) + (6 × IIb) + (0 × IIc) + (100 × surface nodularity) + (78 × surface granularity) + (38 × surface unevenness) + (89 × deep depression) + (100 × thick notch) with 0 when each factor (no) and 1 (yes). This prediction model showed an Area under the curve (AUC) of 0.921 with 95% CI 0.881–0.960 (Figure 1). The best cut-off risk point was 84 and showed a sensitivity of 0.85; specificity, 0.83; positive predictive value, 0.55; negative predictive value, 0.96; and accuracy, 0.83 (Table 3). Three representative examples with images are shown in Figure 2.

**Table 3:** Diagnostic values of the prediction model for beyond SM1 invasion

Cutoff	Accuracy	Sensitivity	Specificity	Positive predictive value	Negative predictive value	Positive by prediction model, *n* (%)
183	0.85	0.23	1.00	1.00	0.84	9 (22.5)
147	0.86	0.30	0.99	0.92	0.85	12 (30.0)
138	0.88	0.43	0.99	0.89	0.88	17 (42.5)
126	0.89	0.53	0.98	0.84	0.89	19 (47.5)
122	0.88	0.53	0.97	0.81	0.89	21 (52.5)
106	0.88	0.55	0.96	0.79	0.90	26 (65.0)
89	0.88	0.68	0.93	0.69	0.92	27 (67.5)
84	0.83	0.85	0.83	0.55	0.96	34 (85.0)
78	0.81	0.88	0.80	0.51	0.96	35 (87.5)
47	0.80	0.90	0.77	0.49	0.97	36 (90.0)
44	0.67	0.98	0.60	0.37	0.99	39 (97.5)
38	0.66	1.00	0.58	0.37	1.00	40 (100.0)

SM1, submucosal invasion < 200 μm.

### Validation of the prediction model for beyond SM1 invasion

**Figure 3:**
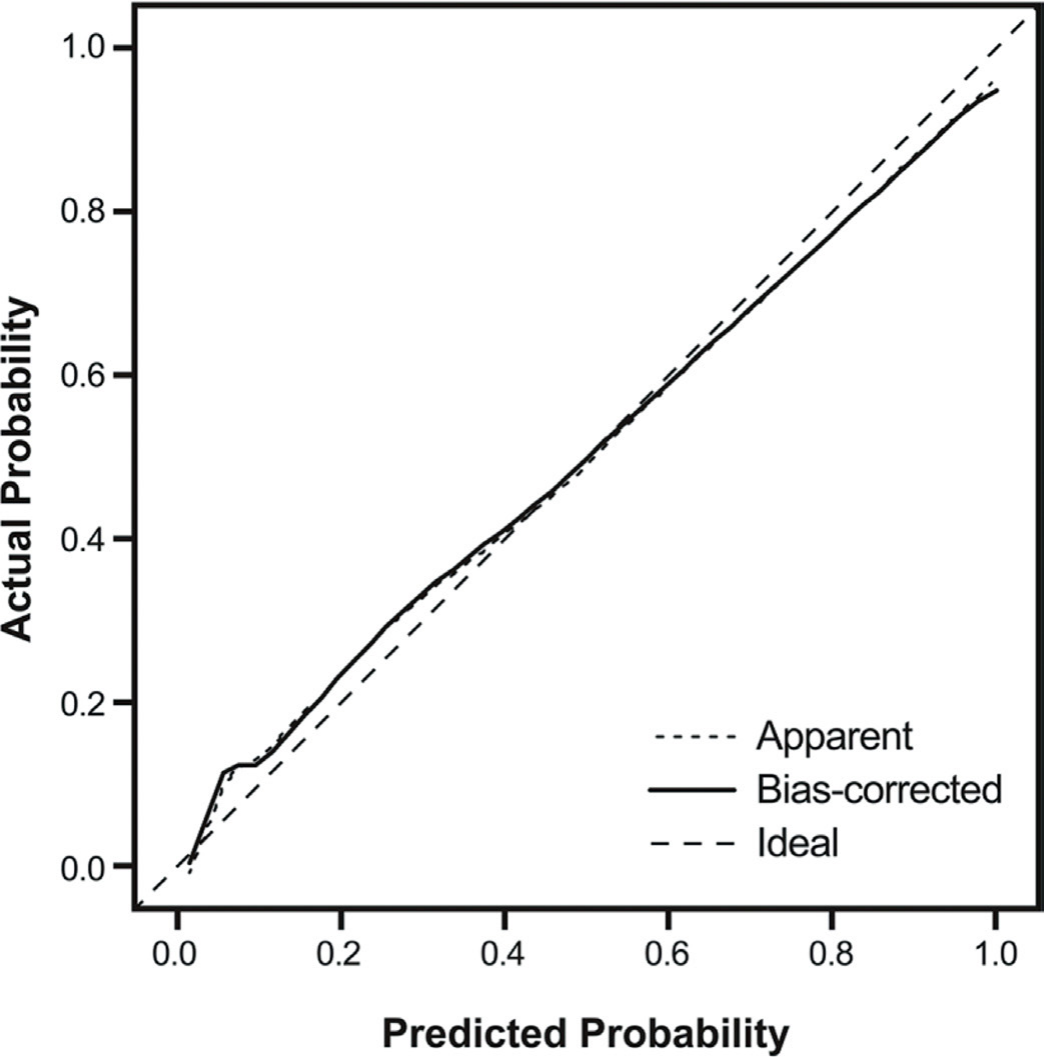
Calibration plot of predicted probabilities and observed probabilities of beyond SM1 invasion in SESCC The C-index was 0.920 (95% CI, 0.874–0.956). SM1, submucosal invasion < 200 μm; SESCC, superficial esophageal squamous cell carcinoma; CI, confidence interval.

The AUC of 10-fold cross validation was 0.889 (95% CI 0.840–0.938). In calibration plots, the apparent line computed with the nomogram and the bias-corrected line computed using 1000 bootstrapping were identical and almost coincided with the ideal line; thus, the prediction power of the nomogram was good (Figure 3).

### Nomogram of prediction for beyond SM1 invasion

**Figure 4:**
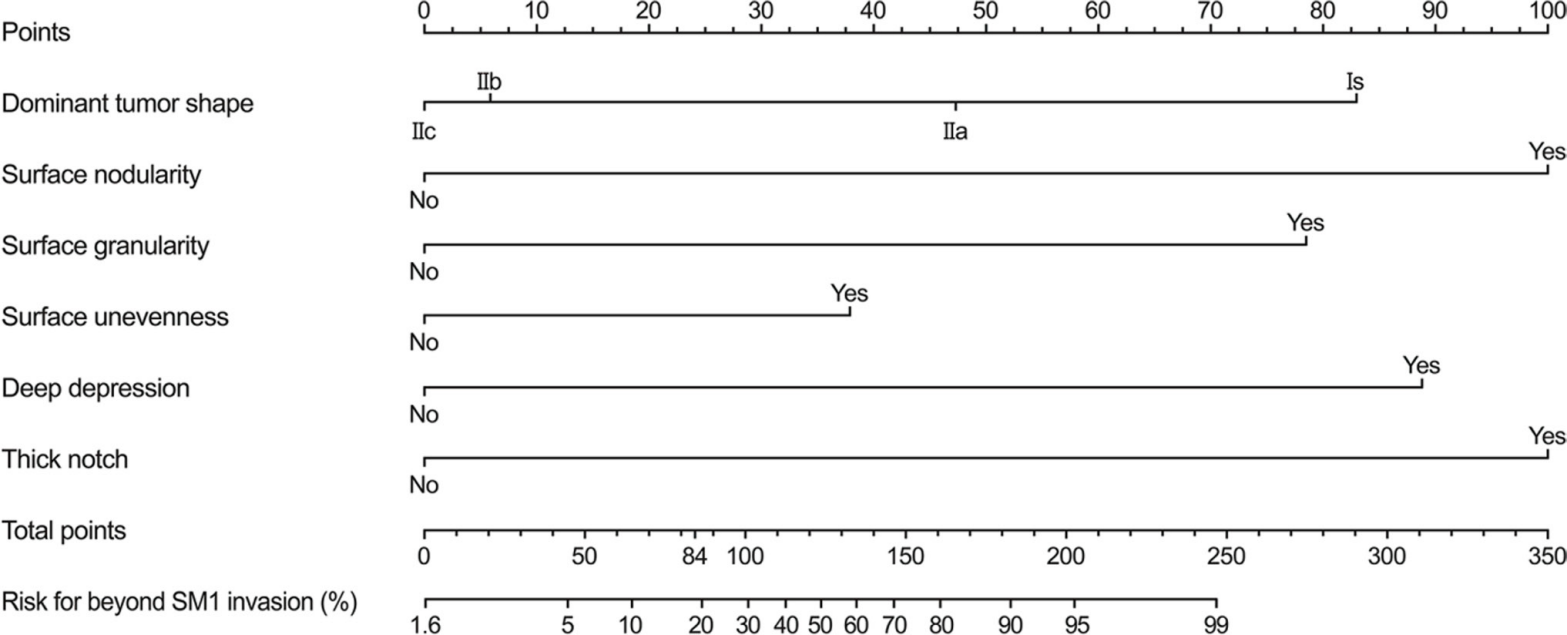
Nomogram of prediction model for beyond SM1 invasion in SESCC Risk points of each endoscopic characteristic in SESCC were identified, and all points were added to calculate total point. A vertical line was drawn at the total point to find the corresponding probability of beyond SM1 invasion. SM1, submucosal invasion < 200 μm; SESCC, superficial esophageal squamous cell carcinoma.

The nomogram for bedside use was developed using the prediction model (Figure 4). In the nomogram, the assigned risk points of each endoscopic characteristic were expressed in the upper straightedge. The probability of beyond SM1 invasion corresponding to each total point was represented by a straightedge at the bottom of the nomogram.

### Inter-observer agreement for endoscopic characteristics

Agreement for assessing endoscopic characteristics which are included in the prediction model was moderate or substantial. Agreement was moderate for thick notch (κ = 0.521) and substantial for main shape, surface nodularity, surface granularity, surface unevenness, and deep depression (κ = 0.794, 0.640, 0.772, 0.693, and 0.605, respectively).

## DISCUSSION

Esophageal ESD is widely performed to treat SESCC because it is less invasive and can preserve organs, unlike surgery [7]. However, due to the risk of LNM, esophageal ESD could be applied as a relative indication in the treatment of SESCC between M3 and SM1 invasion [13]. Therefore, differentiating within SM1 invasion from SM2 invasion is necessary to select proper ESD candidates. Similar to endoscopic ultrasound (EUS) and ME with NBI, white light endoscopy can predict depth of tumor invasion [16, 17, 26–30]. However, the risk estimation from endoscopic findings needs objectivity and should be summed to increase accuracy. We analyzed esophageal ESD cases for SESCC to investigate endoscopic characteristics related to beyond SM1 invasion and create a prediction model differentiating SM1 invasion and beyond in SESCC. Finally, we found endoscopic characteristics associated with beyond SM1 invasion and established a prediction model with excellent discrimination performance.

Protruding lesions or excavated lesions in esophageal neoplastic lesions are associated with SM invasion [25]. However, endoscopic features associated with middle or deeper SM invasion have not been investigated. In the present study, we found certain elevated features and definitely depressed lesions are associated with beyond SM1 invasion. On the other hand, simple IIa and IIc tumor shapes, which have only a slight morphologic change, were not independently associated with beyond SM1 invasion. In addition, one- or two-dimensional tumor sizes were not an independent risk factor for beyond SM1 invasion. Thus, esophageal ESD should be considered with a possible relative indication even when the tumor has a slight morphologic change or a large size to avoid unnecessary surgery. Instead, calculation of the sum of the total risks is important, not the presence or absence of each risk factor. Our prediction model evaluates endoscopic findings objectively and calculates the overall risk of beyond SM1 invasion.

A simple algorithm to determine the treatment direction in patients with SESCC could be suggested using our prediction model. If a patient with SESCC has a total point within 84, esophageal ESD can be performed due to its high negative predictive value to predict beyond SM1 invasion. Following ESD, a precise pathologic examination of the resected specimen can evaluate the curativity, and the necessity and direction of further management considering the patient condition can be determined.

In a recent study, WLI without a mathematical model could distinguish beyond M3 invasion in SESCC with a sensitivity of 0.61 and specificity of 0.77 [17]. However, prediction for beyond SM1 invasion was not evaluated. Only a few studies have predicted beyond SM1 invasion using ME with NBI and EUS [30, 31]. In a prospective study, type B microvessel morphology showed the diagnostic role in predicting depth of tumor invasion of SESCC [30]. Type B1/2 was observed in 9 SESCC beyond SM1 invasions (4.5%, 9/200) although all type B3 was observed in 11 SESCC beyond SM1 invasions (11/11). However, ME requires special equipment and training. Furthermore, the real diagnostic power of ME with NBI remains unclear. ME with NBI showed no additional benefit to WLI for diagnosis of invasion depth of SESCC in a recent study. Thus, ME with NBI could be applied as an additional evaluation after WLE when available [17]. EUS can also predict depth of tumor invasion in SESCC [16, 31]. However, objectively differentiating SM1 invasion from beyond SM1 invasion is difficult.

The current study has some limitations. First, this is a retrospective study in a single center. Thus, a validation in other populations is necessary for generalization. However, ESD is not widely applied for SESCC with a relative indication in Korea, which made it difficult to perform an external validation. Second, an inter- or intra-observer variation may exist in determining endoscopic features. To overcome this, we developed objective criteria, such as classification of nodularity, granularity, and unevenness as protruding lesions of 5 mm or more, 2–5 mm or less, 2 mm or less. Even though some cases cannot be distinguished with respect to deep depression and thick notch, their risk points are similar, and the effects of misclassification on the risk of beyond SM1 invasion may not be profound. Nevertheless, evaluation of inter- or intra-observer agreements in future studies would be desirable. Lastly, we included only ESD cases due to different histologic evaluations in surgical specimen. Our results from the analysis of only ESD specimens may be helpful for endoscopists from a point view of practice. In conclusion, endoscopic features can predict beyond SM1 invasion in SESCC. A mathematical prediction model calculating the sum is a simple, accurate modality to predict beyond SM1 invasion in SESCC.

## MATERIALS AND METHODS

### Study design and population

**Figure 5:**
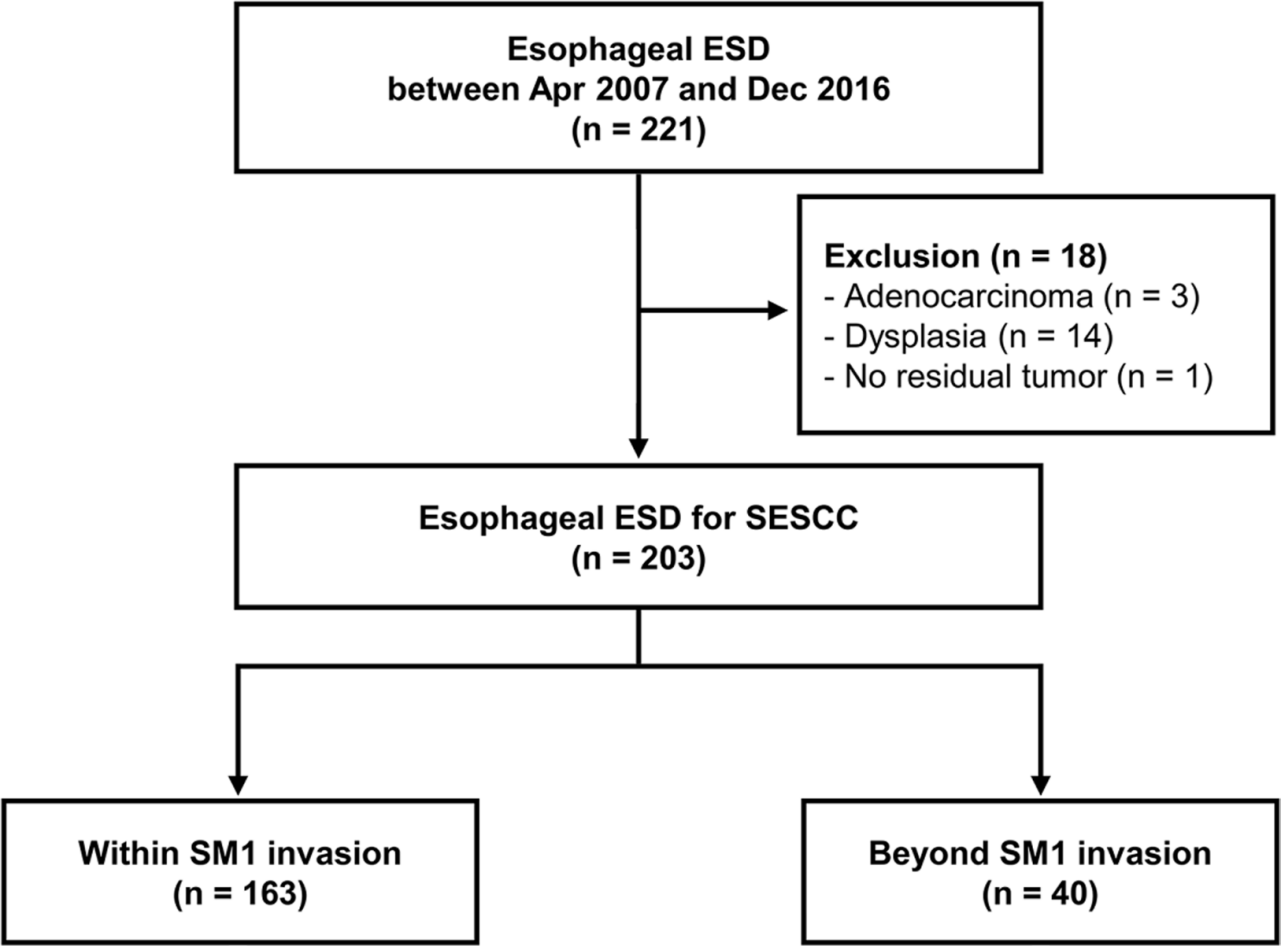
Flow chart of case selection Among 221 cases of esophageal ESDs, which were performed between April 2007 and December 2016, we excluded 18 cases, which were not squamous cell carcinoma. Finally, a total of 203 cases of esophageal ESD were included in this study. Among the 203 cases, 163 were within SM1 invasion and 40 invaded beyond SM1. ESD, endoscopic submucosal dissection; SESCC, superficial esophageal squamous cell carcinoma; SM1, submucosal invasion < 200 μm.

This retrospective study included 221 esophageal ESD cases performed at Samsung Medical Center between April 2007 and December 2016 (Figure 5). Eighteen cases (3 adenocarcinomas, 14 dysplasias, and 1 no remnant tumor after ESD) were excluded from the study. Finally, a total of 203 SESCCs were included in the analysis. In our institution, esophageal ESD was performed when SESCC could be a mucosal cancer or at least SM1 cancer without distant or LNM excluding those with obvious SM invasion. This study protocol was conducted in accordance with the Declaration of Helsinki and was approved by the Institutional Review Board of Samsung Medical Center (No. 2016-11-113-002).

### Data collection

**Figure 6:**
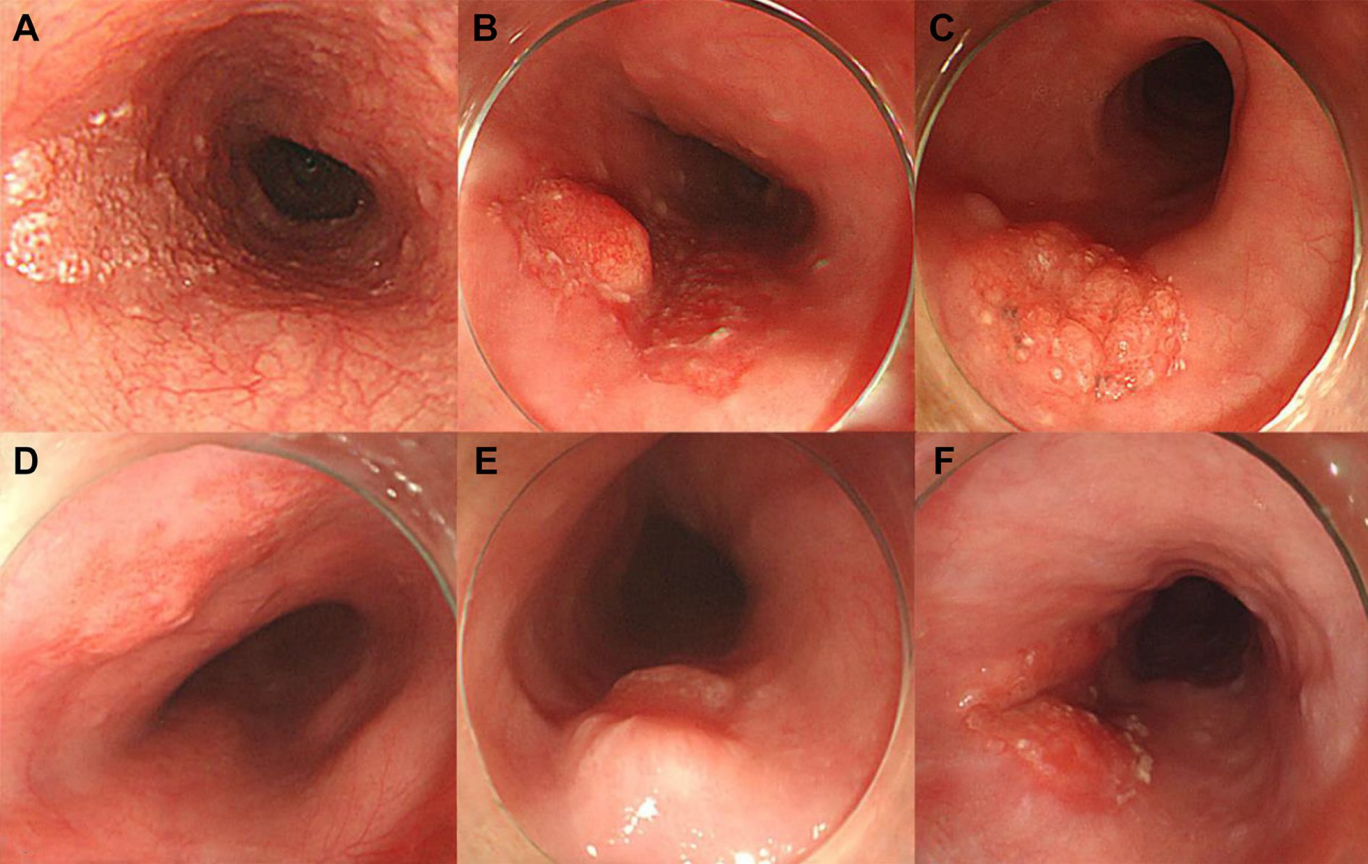
Images of endoscopic tumor characteristics in SESCC (**A**) Flat elevated lesion, type IIa, in the middle of the esophagus. (**B**) Surface nodularity, protuberances > 5 mm on the tumor surface. (**C**) Surface granularity, protuberances between 2 and 5 mm on the tumor surface. (**D**) Surface unevenness, protuberances < 2 mm on the tumor surface. (**E**) Deep depression, obviously deeper than IIc. (**F**) Thick notch, V-shaped notch with surrounding thickened margin. SESCC, superficial esophageal squamous cell carcinoma.

We investigated demographic factors (age, sex, body mass index), tumor characteristics, and final pathologic results of tumor. All endoscopic images were reviewed by two experienced endoscopists who were blinded to the pathologic results. We evaluated endoscopic tumor appearance regarding main shape, sizes (long and short diameter), and surface characteristics. Main shape of tumors was classified as Is, IIa, IIb, and IIc by endoscopic gross configuration (a sessile lesion, Is; a slightly elevated lesion, IIa; a flat lesion, IIb; a slightly depressed lesion, IIc) [25]. Deep depression (deeper than IIc) was defined when evident (Figure 6E). Three types of tumor sizes were measured. First, long tumor size was defined as maximal length of tumor. Second, short tumor size was defined as maximum vertical length of maximum tumor diameter. Lastly, two-dimensional size was obtained by multiplying long tumor size and short tumor size. The surface characteristics were divided into surface erosion, surface nodularity, surface granularity, and surface unevenness. We defined surface nodularity, granularity, and unevenness as gross protuberance size > 5 mm, between 2 and 5 mm, and < 2 mm, respectively (Figure 6B–6D). Information regarding multiplicity of elevated foci and thick notch was collected as another tumor characteristic feature. Multiplicity of elevated foci was defined as more than 3 foci of surface nodularity or granularity. Thick notch was defined as V-shaped notch with surrounding thickened margin (Figure 6F). We obtained data on tumor differentiation, depth of invasion, and presence of LVI through the ESD specimen. Depth of invasion was classified as M1 (limited to the intraepithelium), M2 (limited to the lamina propria), M3 (limited to the muscularis mucosa), SM1 (submucosal invasion < 200 μm), and beyond SM1 (submucosal invasion ≥ 200 μm).

### Statistical analyses

Statistical analyses were performed with SAS version 9.4 (SAS Institute, Cary, NC, USA) and R version 3.3.3 (Vienna, Austria; http://www.R-project.org). Categorical variables were compared using the chi-square test, and continuous data with a normal distribution were compared with the Student t test. Binary multivariate logistic regression analysis with stepwise selection with entry rule 0.2 and stay rule 0.1 was used to identify factors associated with beyond SM1 invasion. All endoscopic characteristics were included in multivariate logistic analysis with stepwise selection to estimate the independent effect of each factor after adjusting for the contributions of other factors. Associations were summarized using ORs and associated 95% CIs for beyond SM1 invasion. Variance inflations of all variables which are included in the model were all less than two indicating that there was no co-linearity between those variables. Models with a C-statistic near 1 demonstrate excellent predictive ability and those near 0.5 demonstrate poor predictive ability. Regression coefficients of multivariate logistic model were used to generate a nomogram predicting the probability of beyond SM1 invasion. Calibration plots were generated to assess the agreement between actual and predicted probabilities for a nomogram. The Bootstrap method was performed to penalize for overfitting of predicted probability. The ability of the prediction model to estimate the risk of beyond SM1 invasion was assessed using the C-statistic and the area under receiver operating characteristics curve value. Tenfold cross validation was used to validate the prediction model. Another endoscopist reviewed all images to assess the agreement using κ coefficient for each endoscopic characteristics which are included in the model. *P* values at the 0.05 level were considered statistically significant.

## Abbreviations

AUC: area under the curve
BMI: body mass index
CI: confidence interval
ESD: endoscopic submucosal dissection
EUS: endoscopic ultrasound
LNM: lymph node metastasis
LVI: lymphovascular invasion
M1: limited to the intraepithelium
M2: limited to the lamina propria
M3: limited to the muscularis mucosa
ME: magnifying endoscopy
NBI: narrow-band imaging
OR: odds ratio
SESCC: superficial esophageal squamous cell carcinoma
SM1: submucosal invasion < 200 μm

## References

[1] Fitzmaurice C, Dicker D, Pain A, Hamavid H, Moradi-Lakeh M, MacIntyre MF, Allen C, Hansen G, Woodbrook R, Wolfe C, Hamadeh RR, Moore A, Werdecker A (2015). The Global Burden of Cancer 2013. JAMA Oncol.

[2] Lee KS, Oh DK, Han MA, Lee HY, Jun JK, Choi KS, Park EC (2011). Gastric cancer screening in Korea: report on the national cancer screening program in 2008. Cancer Res Treat.

[3] Shimizu Y, Takahashi M, Yoshida T, Ono S, Mabe K, Kato M, Asaka M, Sakamoto N (2013). Endoscopic resection (endoscopic mucosal resection/ endoscopic submucosal dissection) for superficial esophageal squamous cell carcinoma: current status of various techniques. Dig Endosc.

[4] Kumagai Y, Monma K, Kawada K (2004). Magnifying chromoendoscopy of the esophagus: in-vivo pathological diagnosis using an endocytoscopy system. Endoscopy.

[5] Yoshida T, Inoue H, Usui S, Satodate H, Fukami N, Kudo SE (2004). Narrow-band imaging system with magnifying endoscopy for superficial esophageal lesions. Gastrointest Endosc.

[6] Tachibana M, Hirahara N, Kinugasa S, Yoshimura H (2008). Clinicopathologic features of superficial esophageal cancer: results of consecutive 100 patients. Ann Surg Oncol.

[7] Ono S, Fujishiro M, Niimi K, Goto O, Kodashima S, Yamamichi N, Omata M (2009). Long-term outcomes of endoscopic submucosal dissection for superficial esophageal squamous cell neoplasms. Gastrointest Endosc.

[8] Ciocirlan M, Lapalus MG, Hervieu V, Souquet JC, Napoleon B, Scoazec JY, Lefort C, Saurin JC, Ponchon T (2007). Endoscopic mucosal resection for squamous premalignant and early malignant lesions of the esophagus. Endoscopy.

[9] Eguchi T, Nakanishi Y, Shimoda T, Iwasaki M, Igaki H, Tachimori Y, Kato H, Yamaguchi H, Saito D, Umemura S (2006). Histopathological criteria for additional treatment after endoscopic mucosal resection for esophageal cancer: analysis of 464 surgically resected cases. Mod Pathol.

[10] Akutsu Y, Uesato M, Shuto K, Kono T, Hoshino I, Horibe D, Sazuka T, Takeshita N, Maruyama T, Isozaki Y, Akanuma N, Matsubara H (2013). The overall prevalence of metastasis in T1 esophageal squamous cell carcinoma: a retrospective analysis of 295 patients. Ann Surg.

[11] Choi JY, Park YS, Jung HY, Ahn JY, Kim MY, Lee JH, Choi KS, Kim DH, Choi KD, Song HJ, Lee GH, Cho KJ, Kim JH (2011). Feasibility of endoscopic resection in superficial esophageal squamous carcinoma. Gastrointest Endosc.

[12] Pimentel-Nunes P, Dinis-Ribeiro M, Ponchon T, Repici A, Vieth M, De Ceglie A, Amato A, Berr F, Bhandari P, Bialek A, Conio M, Haringsma J, Langner C (2015). Endoscopic submucosal dissection. European Society of Gastrointestinal Endoscopy (ESGE) Guideline. Endoscopy.

[13] Japan Esophageal Society (2017). Japanese Classification of Esophageal Cancer. part I. Esophagus.

[14] Higuchi K, Tanabe S, Koizumi W, Sasaki T, Nakatani K, Saigenji K, Kobayashi N, Mitomi H (2007). Expansion of the indications for endoscopic mucosal resection in patients with superficial esophageal carcinoma. Endoscopy.

[15] Takahashi H, Arimura Y, Masao H, Okahara S, Tanuma T, Kodaira J, Kagaya H, Shimizu Y, Hokari K, Tsukagoshi H, Shinomura Y, Fujita M (2010). Endoscopic submucosal dissection is superior to conventional endoscopic resection as a curative treatment for early squamous cell carcinoma of the esophagus (with video). Gastrointest Endosc.

[16] Thosani N, Singh H, Kapadia A, Ochi N, Lee JH, Ajani J, Swisher SG, Hofstetter WL, Guha S, Bhutani MS (2012). Diagnostic accuracy of EUS in differentiating mucosal versus submucosal invasion of superficial esophageal cancers: a systematic review and meta-analysis. Gastrointest Endosc.

[17] Ebi M, Shimura T, Yamada T, Mizushima T, Itoh K, Tsukamoto H, Tsuchida K, Hirata Y, Murakami K, Kanie H, Nomura S, Iwasaki H, Kitagawa M (2015). Multicenter, prospective trial of white-light imaging alone versus white-light imaging followed by magnifying endoscopy with narrow-band imaging for the real-time imaging and diagnosis of invasion depth in superficial esophageal squamous cell carcinoma. Gastrointest Endosc.

[18] Yoon YC, Lee KS, Shim YM, Kim BT, Kim K, Kim TS (2003). Metastasis to regional lymph nodes in patients with esophageal squamous cell carcinoma: CT versus FDG PET for presurgical detection prospective study. Radiology.

[19] Ra J, Paulson EC, Kucharczuk J, Armstrong K, Wirtalla C, Rapaport-Kelz R, Kaiser LR, Spitz FR (2008). Postoperative mortality after esophagectomy for cancer: development of a preoperative risk prediction model. Ann Surg Oncol.

[20] Chang AC, Ji H, Birkmeyer NJ, Orringer MB, Birkmeyer JD (2008). Outcomes after transhiatal and transthoracic esophagectomy for cancer. Ann Thorac Surg.

[21] Connors RC, Reuben BC, Neumayer LA, Bull DA (2007). Comparing outcomes after transthoracic and transhiatal esophagectomy: a 5-year prospective cohort of 17,395 patients. J Am Coll Surg.

[22] Park HC, Kim DH, Gong EJ, Na HK, Ahn JY, Lee JH, Jung KW, Choi KD, Song HJ, Lee GH, Jung HY, Kim JH (2016). Ten-year experience of esophageal endoscopic submucosal dissection of superficial esophageal neoplasms in a single center. Korean J Intern Med.

[23] Tsujii Y, Nishida T, Nishiyama O, Yamamoto K, Kawai N, Yamaguchi S, Yamada T, Yoshio T, Kitamura S, Nakamura T, Nishihara A, Ogiyama H, Nakahara M (2015). Clinical outcomes of endoscopic submucosal dissection for superficial esophageal neoplasms: a multicenter retrospective cohort study. Endoscopy.

[24] Yamashina T, Ishihara R, Nagai K, Matsuura N, Matsui F, Ito T, Fujii M, Yamamoto S, Hanaoka N, Takeuchi Y, Higashino K, Uedo N, Iishi H (2013). Long-term outcome and metastatic risk after endoscopic resection of superficial esophageal squamous cell carcinoma. Am J Gastroenterol.

[25] Endoscopic Classification Review Group (2005). Update on the paris classification of superficial neoplastic lesions in the digestive tract. Endoscopy.

[26] Pech O, Gunter E, Dusemund F, Origer J, Lorenz D, Ell C (2010). Accuracy of endoscopic ultrasound in preoperative staging of esophageal cancer: results from a referral center for early esophageal cancer. Endoscopy.

[27] Chemaly M, Scalone O, Durivage G, Napoleon B, Pujol B, Lefort C, Hervieux V, Scoazec JY, Souquet JC, Ponchon T (2008). Miniprobe EUS in the pretherapeutic assessment of early esophageal neoplasia. Endoscopy.

[28] Esaki M, Matsumoto T, Moriyama T, Hizawa K, Ohji Y, Nakamura S, Hirakawa K, Hirahashi M, Yao T, Iida M (2006). Probe EUS for the diagnosis of invasion depth in superficial esophageal cancer: a comparison between a jelly-filled method and a water-filled balloon method. Gastrointest Endosc.

[29] Goda K, Tajiri H, Ikegami M, Yoshida Y, Yoshimura N, Kato M, Sumiyama K, Imazu H, Matsuda K, Kaise M, Kato T, Omar S (2009). Magnifying endoscopy with narrow band imaging for predicting the invasion depth of superficial esophageal squamous cell carcinoma. Dis Esophagus.

[30] Oyama T, Inoue H, Arima M, Momma K, Omori T, Ishihara R, Hirasawa D, Takeuchi M, Tomori A, Goda K (2017). Prediction of the invasion depth of superficial squamous cell carcinoma based on microvessel morphology: magnifying endoscopic classification of the Japan Esophageal Society. Esophagus.

[31] Ishihara R, Matsuura N, Hanaoka N, Yamamoto S, Akasaka T, Takeuchi Y, Higashino K, Uedo N, Iishi H (2017). Endoscopic imaging modalities for diagnosing invasion depth of superficial esophageal squamous cell carcinoma: a systematic review and meta-analysis. BMC Gastroenterol.

